# Illicit drug use and its association with key sexual risk behaviours and outcomes: Findings from Britain’s third National Survey of Sexual Attitudes and Lifestyles (Natsal-3)

**DOI:** 10.1371/journal.pone.0177922

**Published:** 2017-05-18

**Authors:** Rachelle Paquette, Clare Tanton, Fiona Burns, Philip Prah, Maryam Shahmanesh, Nigel Field, Wendy Macdowall, Kirsten Gravningen, Pam Sonnenberg, Catherine H. Mercer

**Affiliations:** 1 Research Department of Infection and Population Health, University College London, London, United Kingdom; 2 Department of Social and Environmental Health Research, London School of Hygiene and Tropical Medicine, London, United Kingdom; 3 Department of Microbiology and Infection Control, University Hospital of North Norway, Tromsø, Norway; Universita Cattolica del Sacro Cuore Sede di Roma, ITALY

## Abstract

**Objectives:**

We explore the hypothesis that using illicit drugs other than, or in addition to, cannabis is associated with sexual risk behaviour and sexual health outcomes in the British population.

**Methods:**

We analysed data, separately by gender, reported by sexually-active participants (those reporting > = 1 partners/past year) aged 16–44 years (3,395 men, 4,980 women) in Britain’s third National Survey of Sexual Attitudes and Lifestyles (Natsal-3), a probability survey undertaken 2010–12 involving computer-assisted personal-interview and computer-assisted self-interview. Analyses accounted for the stratification, clustering and weighting of the data. Multivariable logistic regression was used to calculate adjusted odds ratios.

**Results:**

Use of illicit drugs other than, or in addition to, cannabis in the past year was reported by 11.5% (95%CI:10.4%-12.8%) of men and 5.5% (4.8%-6.3%) of women. Use of these types of drugs was more common among those <35 years, those who reported poor general and/or sexual health behaviours, e.g. binge drinking > = weekly (age-adjusted ORs, aAORs, 10.91 (6.27–18.97) men; 9.95 (6.11–16.19) women); having > = 2 condomless partners in the past year (aAOR:5.50 (3.61–8.39) men; 5.24 (3.07–8.94) women). Participants reporting illicit drug use were more likely (than those who did not) to report sexual health clinic attendance (ORs after adjusting for age, sexual identity and partner numbers: 1.79 (1.28–2.51) men; 1.99 (1.34–2.95) women), chlamydia testing (1.42 (1.06–1.92) men; 1.94 (1.40–2.70) women), unplanned pregnancy (2.93 (1.39–6.17) women), and among men only, sexually transmitted infection diagnoses (3.10 (1.63–5.89)).

**Conclusions:**

In Britain, those reporting recent illicit drug use were more likely to report other markers of poor general and sexual health. They were also more likely to attend sexual health clinics so these should be considered appropriate settings to implement holistic interventions to maximise health gain.

## Introduction

In 2013, 3.4–6.6% of the world’s population aged 15–64 years were estimated to have engaged in illicit drug use.[1] In England and Wales in 2013/14, 8.8% of 16–59 year olds (approximately 2.7 million people) had used an illicit drug in the past year, and among 16–24 year olds, the prevalence was twice as high at 18.9%.[2] Use was also higher in men who have sex with men (MSM) and women who have sex with women (WSW).[2]

Despite reductions in sexual risk behaviour observed at a population level in Britain, [3] there has not been a decline in sexually transmitted infections (STIs) [4] and it is estimated that one in six pregnancies are unplanned.[5] Continued efforts to understand the factors that shape sexual health risk are needed, and in recent times the focus has shifted to contextual factors including illicit drug use. For example, studies report an association between drug use and sexual risk behaviours[6–8] such as having multiple sexual partners and condomless sex with casual partners.[9–12] Associations have also been observed between illicit drug use and adverse sexual health outcomes e.g. STI diagnoses.[13–15]

The mechanisms through which drug use may impact on sexual risk behaviour and adverse sexual health outcomes include the situational effect of drugs on decision-making [16] whereby an individual may engage in sexual activity that was not intended prior to the consumption of drugs. Alternative mechanisms include the clustering of behaviours due to personality factors or other underlying risks. Studies have also highlighted associations between drug use and a higher prevalence of health-limiting behaviours such as cigarette smoking, and binge-drinking[17] and point to a clustering of risk with depressive symptoms.[9,10,14,18–21] In MSM, this clustering of poor mental, sexual and general health and behaviours leads to an increased risk of adverse sexual health outcomes, [22] suggesting that understanding such mechanisms requires a syndemic approach.[23]

There is a need for a population perspective, as current research has largely focused on sub-groups such as MSM, [9,10,13,14,17,18,20] or, if conducted in the general population, has tended to focus on cannabis use in young people.[7,11,12] While cannabis remains the most commonly used illicit drug, [2] club drug (drugs whose use is typically associated with dance parties and nightclubs) [2] use is also high in Britain. [2,8] In this paper we explore the hypothesis that using illicit drugs is associated with sexual risk behaviours and adverse sexual health outcomes in the British general population using data from a recent probability sample survey. We start by presenting prevalence estimates of recent use of different types of drugs, including and also excluding cannabis. We then focus on the reporting of use of drugs other than, or in addition to, cannabis as a marker of (harder) drug use and to address the gap in the literature. We explore how the prevalence of this measure (use of drugs other than, or in addition to, cannabis) varies by sociodemographic and health-related factors and also sexual behaviours to explore whether associations and clustering observed in smaller sub-populations (e.g. MSM) are seen in the general population. Finally, we examine whether reporting key sexual health outcomes varies according to whether use of drugs other than, or in addition to, cannabis was reported, after accounting for key influences.

## Methods

### Data

Full methodological details of Britain’s third National Survey of Sexual Attitudes and Lifestyles (Natsal-3) have previously been published, and are available from the study’s website: www.natsal.ac.uk, including the Natsal-3 questionnaire.[24,25] Briefly, Natsal-3 was a complex multi-staged survey involving a stratified, clustered probability sample design. Altogether, 15,162 interviews with people aged 16–74 years resident in Britain were completed between September 2010 and August 2012. The response rate was 57.7%. Computer-assisted personal-interviewing (CAPI) was used, with computer-assisted self-interview (CASI) for the more sensitive questions. Of relevance to this paper, questions about the participant’s general health were asked in an initial face-to-face interview using showcards. The more sensitive questions, including those on sexual behaviours and experience of key sexual health outcomes were asked in the CASI. The questions about illicit drug use were also asked in the CASI beginning: “Have you **ever** taken any of the drugs listed below? (Please **do not** count any drugs you have injected).” The response options were worded:

Cannabis (marijuana, grass, hash, ganja, draw, skunk, weed, spliff)
Amphetamines (speed, whizz, uppers, billy)
Cocaine or coke (charlie)
Crack (rock, stones, white)
Ecstasy (E)
Heroin that was not injected (smack, skag, H, brown, gear, horse)
Acid or LSD (tabs, trips) or magic mushrooms
Crystal Meth
Amyl Nitrates (poppers, liquid gold, rush)
Other non-prescribed drugs

Those reporting ever use of cannabis were then asked “Have you taken cannabis in the last 12 months?” Those reporting ever use of the remaining drugs (with the exception of ‘other non-prescribed drugs’) were then asked “You mentioned that you had taken (name of drug/s). Have you taken (this drug/any of these drugs) in the last 12 months?” If so, participants were asked whether they had done so in the past 4 weeks. We focus our analyses on use in the past year as a compromise between ever use, which is more likely to capture transient experiences, and use in the past four weeks, which would reduce statistical power. The timeframe of the past year also has the advantage that it tallies with the timeframe of many of the behaviours that Natsal-3 asked about.

### Statistical analyses

We used the complex survey functions of Stata V.13.1 (StataCorp LP, College Station, Texas) for all statistical analyses to take account of the stratification, weighting and clustering of the Natsal-3 data. The data were weighted to adjust for the unequal probabilities of selection and for non-response. We restricted our analysis to 16–44 year olds, as among older people there was relatively low prevalence of reporting of recent illicit drug use and of our outcomes of interest[4] We limited our denominator to the ‘sexually-active’ population (those reporting at least one partner in the past year) as a key objective of the paper is to understand the association between drug use and sexual behaviour and experience of sexual health outcomes. Throughout we consider men and women separately recognising the gender differences in reporting of illicit drug use, as well as in the experience and reporting of sexual behaviours, [3] and the ‘sexual scripts’ which shape these behaviours.[26,27]

We first estimated the prevalence and corresponding 95% confidence interval (CI) of reporting illicit drug use (excluding injected illicit drugs) in the past year, which we stratified according to:

1. Only cannabis
2. Both cannabis and other illicit drugs
3. Only illicit drugs other than cannabis

As Natsal-3 did not ask about which particular drug(s) had been used in the past year it is not possible to give drug-specific prevalence estimates for the timeframe of interest in this paper, but for context, we provide estimates of ever use of the specific drugs for men and women in S1 Web-Appendices A and B, respectively.

We created a binary variable ‘illicit drug use other than, or in addition to, cannabis in the past year’ (categories 2 and 3 above) vs. only cannabis / no illicit drug use, and used this in our bivariate and multivariable analyses. We focus on illicit drugs that were not injected reflecting the wording of our question. Moreover, the prevalence of injecting drug use (asked in a separate question) was low with only 0.4% of men and 0.1% of women reporting having injected drugs in the past year, and only 1.5% and 1.8% of those men and women who reported using drugs other than, or in addition to, cannabis in the past year. Treating our measure of illicit drug use firstly as a dependent (outcome) variable, we considered how the prevalence varied according to sociodemographic and health-related factors and sexual behaviours (question wording can be found in the Natsal questionnaire at www.natsal.ac.uk). We used logistic regression to calculate crude odds ratios (OR) as well as age-adjusted ORs (aAORs) to control for the potential confounding effect of age on the association between each of the independent variables considered and illicit drug use. We then treated illicit drug use as an independent variable and used multivariable logistic regression to examine how the reporting of key sexual health outcomes varies according to whether or not men and women reported illicit drug use. Sexual health outcomes (all in the past year) were: attendance at a sexual health (GUM) clinic; diagnosis with STI(s) (chlamydia, gonorrhoea, genital warts, trichomonas vaginalis, syphilis, Non Specific Urethritis / Non Gonococcal Urethritis (men only), herpes); being tested for HIV; being tested for chlamydia; and, for women, unplanned pregnancy. Unplanned pregnancy was defined according to the psychometrically validated London Measure of Unplanned Pregnancy (LMUP) which comprises 6 questions asking about contraceptive use, timing of motherhood, intention to become pregnant, desire for a baby, discussion with a partner, and preconceptual preparations. [28,29] We present two models for each outcome, the first includes age along with drug use, to account for the confounding effect of age. Additionally, in order to assess the ‘independent’ association between illicit drug use and our sexual health outcomes, we included age, sexual identity and the number of partners reported in the past year in each model as these factors are strongly associated with both illicit drug use (the hypothesised explanatory variable here) and our outcomes of interest.[30–32]

### Ethics statement

All Natsal-3 participants were given an information leaflet to read prior to participation. In line with standard practice for UK surveys, and in response to evidence suggesting that signing a consent form might lead to a greater sense of obligation to complete the interview, we obtained verbal rather than written consent.[33] We ensured procedures for obtaining verbal informed consent via our interviewer training and protocols: interviewers were trained to make sure that participants had read the information leaflet and had the opportunity to discuss the study fully before the interview began; and at the beginning of each interview, interviewers were prompted (on screen) to remind participants that they could choose not to answer any question. Interviewers had to confirm in the computer programme that respondents had read the information leaflet before commencing the interview. The Natsal-3 study, was approved by the Oxfordshire Research Ethics Committee A (reference: 09/H0604/27). All participants provided their own consent to participate, however for 16–17 year olds living at home, a parent/guardian provided additional verbal assent for participation.

### Data availability

An anonymised dataset is available to academic researchers from the UK Data Service, https://discover.ukdataservice.ac.uk/; SN: 7799; persistent identifier: 10.5255/UKDA-SN-77991-1.

## Results

### The prevalence of recent illicit drug use among sexually-active people aged 16–44 years

Overall, 25.6% of men and 12.5% of women reported having used any of the illicit drugs Natsal-3 asked about in the past year, including cannabis (Table 1). However, among both genders prevalence declined with age from 40.2% of men aged 16–24 years to 14.7% of men aged 35–44 years, and from 21.5% to 5.6%, respectively, among women. Less than half of these men and women reported using drugs other than, or in addition to, cannabis (shown in bold in Table 1). This corresponds to 11.5% of all men 16–44 years and 5.5% of all women 16–44 years and is the focus of our analyses hereon.

**Table 1:** Prevalence (95% CI) of illicit drug use^1^ in the past year, reported by sexually-active people aged 16–44 years in Britain by type of drugs used, gender and age-group.

	Of all sexually-active					Of those reporting any illicit drug use^2^:			
Age (years)	Any illicit drug use^3^	Only cannabis	Both cannabis & other drugs^2^^,^^4^	Drugs other than cannabis^2^^,^^4^	*Denominators*^3^	Only cannabis	Both cannabis & other drugs^2^^,^^4^	Drugs other than cannabis^2^^,^^4^	*Denominators*^3^
	% (95% CI)	% (95% CI)	% (95% CI)	% (95% CI)		% (95% CI)	% (95% CI)	% (95% CI)	
**Men**									
**16–24**	40.2% [37.2–43.3]	25.2% [22.5–28.1]	**11.8%** **[9.9–13.9]**	**3.3%** **[2.3–4.7]**	*1296*, *948*	62.6% [57.7–67.4]	29.3% [25.0–33.9]	8.1% [5.7–11.5]	*516*, *381*
**25–34**	26.0% [23.4–28.8]	12.5% [10.6–14.6]	**8.4%** **[6.8–10.2]**	**4.9%** **[3.8–6.3]**	*1378*, *1238*	48.5% [42.4–54.6]	32.5% [27.2–38.2]	19.1% [15.1–23.7]	*361*, *319*
**35–44**	14.7% [12.1–17.7]	7.3% [5.5–9.5]	**3.7%** **[2.4–5.7]**	**3.6%** **[2.3–5.5]**	*721*, *1302*	50.0% [39.8–60.1]	25.6% [17.3–36.2]	24.4% [16.2–35.1]	*112*, *189*
**All**	25.6% [24.0–27.4]	14.0% [12.7–15.3]	**7.6%** **[6.6–8.6]**	**4.0%** **[3.2–4.8]**	*3395*, *3488*	54.9% [51.3–58.4]	29.6% [26.3–33.2]	15.5% [12.8–18.7]	*989*, *889*
**Women**									
**16–24**	21.5% [19.3–23.9]	13.0% [11.3–15.0]	**6.0%** **[4.8–7.4]**	**2.5%** **[1.8–3.5]**	*1678*, *931*	60.7% [54.8–66.3]	27.8% [22.8–33.4]	11.6% [8.4–15.8]	*362*, *200*
**25–34**	13.0% [11.4–14.8]	6.0% [5.0–7.2]	**3.9%** **[3.0–5.0]**	**3.1%** **[2.4–4.0]**	*2245*, *1250*	46.1% [39.8–52.6]	30.0% [24.3–36.4]	23.9% [18.8–29.8]	*292*, *163*
**35–44**	5.6% [4.2–7.4]	3.6% [2.6–4.9]	**0.7%** **[0.3–1.4]**	**1.3%** **[0.7–2.5]**	*1057*, *1299*	63.8% [50.5–75.3]	12.4% [6.2–23.4]	23.8% [13.7–38.1]	*61*, *73*
**All**	12.5% [11.5–13.7]	7.0% [6.2–7.8]	**3.3%** **[2.8–3.8]**	**2.3%** **[1.8–2.9]**	*4980*, *3481*	55.8% [51.6–59.9]	26.0% [22.7–29.8]	18.2% [14.9–22.0]	*715*, *436*

^1^Excludes injected illicit drugs

^2^The question about illicit drug use asked about specifically: Cannabis (marijuana, grass, hash, ganja, draw, skunk, weed, spliff); Amphetamines (speed, whizz, uppers, billy); Cocaine or coke (charlie); Crack (rock, stones, white); Ecstasy (E); Heroin that was not injected (smack, skag, H, brown, gear, horse); Acid or LSD (tabs, trips) or magic mushrooms; Crystal Meth; Amyl Nitrates (poppers, liquid gold, rush); ‘Other non-prescribed drugs’.

^3^Unweighted, weighted denominators

^4^The two categories used to define the dependent (outcome) variable in Tables 2 and 3 and the hypothesized independent (explanatory) variable in Table 4, defined as using drugs other than, or in addition to, cannabis

### Variations in the prevalence of recent illicit drug use by sociodemographic and health-related factors, and sexual behaviours

As well as the variation by age-group described above, prevalence of reporting illicit drug use other than, or in addition to, cannabis also differed by a number of key sociodemographic and, health-related factors and sexual behaviours (Tables 2 and 3). Among men, those who identified as gay were more likely to report drug use than those identifying as heterosexual (aAOR 6.93), while among women those who identified as bisexual were more likely to do so (aAOR 3.28 for women identifying as bisexual vs. those identifying as heterosexual). Men and women who were married at interview were least likely to report our measure of illicit drug use, including after adjusting for age. Prevalence was higher in those of white or mixed ethnicity than among other ethnicities. In women, but not men, associations were observed for academic qualifications, however, this reflects the confounding effect of age as shown in Table 3.

**Table 2:** Variations in the prevalence of reporting recent illicit drug use^1^ by sociodemographic and health-related factors, and sexual behaviours: Sexually-active men aged 16–44 years.

	Prevalence, %, of reporting recent illicit drug use^1^ (95% CI)	*Crude odds ratio (95% CI)*	*Age-adjusted odds ratio (95% CI)*	Denominators^2^
All sexually-active men aged 16–44 years	11.5% [10.4%-12.8%]	-	-	3395, 3488
***Sociodemographic factors***				
Age		*p<0*.*001*		
16–24	15.0% [12.9%-17.4%]	*1*.*00*		1296, 948
25–34	13.3% [11.3%-15.5%]	*0*.*87* *(0*.*66–1*.*13)*		1378, 1238
35–44	7.3% [5.4%-9.7%]	*0*.*44* *(0*.*31–0*.*63)*		721, 1302
Sexual identity^3^		*p<0*.*001*	*p<0*.*001*	
Heterosexual	10.8% [9.7%-12.0%]	*1*.*00*	*1*.*00*	3274, 3382
Gay	45.9% [32.9%-59.5%]	*7*.*03* *(3*.*97–12*.*45)*	*6*.*93* *(3*.*69–13*.*00)*	79, 66
Bisexual	14.5% [5.5%-33.3%]	*1*.*41* *(0*.*48–4*.*16)*	*1*.*32* *(0*.*45–3*.*85)*	33, 34
Relationship status		*p<0*.*001*	*p<0*.*001*	
Married	5.0% [3.5%-7.1%]	*1*.*00*	*1*.*00*	910, 1380
Cohabitating	15.0% [12.1%-18.6%]	*3*.*35* *(2*.*13–5*.*28)*	*3*.*33* *(2*.*06–5*.*37)*	590, 645
Previously married	11.7% [6.4%-20.3%]	*2*.*50* *(1*.*19–5*.*25)*	*2*.*50* *(1*.*19–5*.*27)*	110, 101
Single/never married	16.4% [14.5%-18.5%]	*3*.*72* *(2*.*49–5*.*55)*	*3*.*66* *(2*.*31–5*.*82)*	1783, 1359
Ethnicity		*p<0*.*001*	*p<0*.*001*	
White	12.7% [11.3%-14.1%]	*1*.*00*	*1*.*00*	2974, 2974
Mixed	15.6% [9.0%-25.6%]	*1*.*27* *(0*.*67–2*.*43)*	*1*.*15* *(0*.*61–2*.*18)*	81, 80
Asian/Asian British	3.1% [1.4%-6.7%]	*0*.*22* *(0*.*96–0*.*51)*	*0*.*24* *(0*.*10–0*.*54)*	174, 246
Black/Black British	2.4% [0.7%-8.0%]	*0*.*17* *(0*.*05–0*.*60)*	*0*.*17* *(0*.*05–0*.*60)*	117, 132
Chinese/other^4^	2.6% [0.8%-8.2%]	*0*.*18* *(0*.*05–0*.*60)*	*0*.*18* *(0*.*06–0*.*60)*	45, 53
Academic qualifications^5^		*p* = 0.125	*p* = 0.565	
None	9.4% [6.6%-13.2%]	1.00	1.00	299, 332
Qualifications typically gained at age 16	10.8% [9.0%-12.8%]	1.16 (0.76–1.79)	1.12 (0.73–1.72)	1165, 1202
Studying for/attained further academic qualifications	12.9% [11.2%-14.8%]	1.43 (0.94–2.17)	1.23 (0.1–1.89)	1745, 1796
***Health-related factors***				
Self-reported health status		*p* = 0.407	*p* = 0.181	
Very good	10.7% [9.0%-12.7%]	1.00	1.00	1602, 1630
Good	12.2% [10.5%-14.3%]	1.17 (0.89–1.53)	1.23 (0.93–1.62)	1419, 1493
Fair	11.4% [8.2%-15.7%]	1.08 (0.70–1.66)	1.15 (0.75–1.78)	320, 317
Bad/very bad	17.6% [9.2%-31.0%]	1.79 (0.83–3.87)	2.14 (0.99–4.65)	54, 48
Treated for depression in the past year		*p* = 0.041	*p* = 0.009	
No	11.3% [10.1%-12.6%]	1.00	1.00	3282, 3381
Yes	18.3% [11.7%-27.4%]	1.76 (1.02–3.02)	2.07 (1.20–3.57)	111, 105
Current smoking status		*p*<0.001	*p*<0.001	
No	7.7% [6.5%-9.2%]	1.00	1.00	2210, 2355
Yes	19.3% [17.0%-21.9%]	2.85 (2.22–3.66)	2.72 (2.12–3.49)	1185, 1133
Current frequency of binge drinking^6^		*p*<0.001	*p*<0.001	
Never	2.7% [1.7%-4.5%]	1.00	1.00	772, 880
Less than monthly	7.9% [6.0%-10.4%]	3.06 (1.69–5.54)	2.91 (1.61–5.28)	860, 884
Monthly	15.6% [12.7%-18.9%]	6.54 (3.71–11.54)	6.20 (3.50–10.97)	739, 716
Weekly/daily	24.6% [21.3%-28.2%]	11.6 (6.69–20.09)	10.9 (6.27–18.97)	814, 782
***Sexual behaviours***				
Number of sexual partners^7^, past year		*p*<0.001	*p*<0.001	
1	8.4% [7.2%-9.7%]	1.00	1.00	2323, 2613
2	15.9% [12.1%-20.7%]	2.07 (1.44–3.00)	1.84 (1.27–2.66)	437, 370
3–4	18.5% [14.3%-23.4%]	2.48 (1.75–3.50)	2.22 (1.56–3.15)	358, 292
5+	34.7% [28.2%-41.7%]	5.80 (4.15–8.10)	5.03 (3.51–7.20)	257, 196
Same-sex partner(s), past year		*p*<0.001	*p*<0.001	
No	10.8% [9.7%-12.1%]	1.00	1.00	3282, 3396
Yes	36.5% [26.1%-48.3%]	4.73 (2.85–7.88)	4.56 (2.63–7.89)	113, 92
Number of partnerships^7^ without a condom, past year		*p*<0.001	*p*<0.001	
0	6.5% [4.7%-9.0%]	1.00	1.00	680, 614
1	9.8% [8.5%-11.3%]	1.57 (1.06–2.33)	1.85 (1.23–2.76)	2129, 2397
2+	27.8% [23.5%-32.4%]	5.53 (3.64–8.41)	5.50 (3.61–8.39)	522, 418
Paid for sex^7^, past year		*p* = 0.019	*p* = 0.010	
No	11.4% [10.2%-12.6%]	1.00	1.00	3351, 3446
Yes	23.2% [12.9%-38.2%]	2.36 (1.15–4.85)	2.47 (1.24–4.94)	44, 42
Used internet to find a sexual partner^7^ past year		*p*<0.001	*p*<0.001	
No	10.8% [9.7%-12.1%]	1.00	1.00	3108, 3247
Yes	20.7% [15.5%-27.1%]	2.15 (1.48–3.12)	2.08 (1.42–3.06)	286, 239

p-values from Wald test

^1^‘Recent illicit drug use’ defined as using drugs other than, or in addition to, cannabis in the past year. Excludes injected drugs.

^2^Unweighted, weighted denominators defined as participants aged 16–44 years reporting at least one sexual partner in the past year.

^3^Excludes those reporting ‘other’ to the question about sexual identity as this was reported by too few participants to provide robust estimates.

^4^‘Chinese’ and ‘other’ subcategories were merged because of small numbers in these categories.

^5^Limited to those aged at least 17 years.

^6^More than 8 units of alcohol on one occasion.

^7^Opposite-sex and/or same-sex sex partner(s).

**Table 3:** Variations in the prevalence of reporting recent illicit drug use by sociodemographic and health-related factors, and sexual behaviours: Sexually-active women aged 16–44 years.

	Prevalence, %, of reporting recent illicit drug use^1^ (95% CI)	Crude odds ratio (95% CI)	Age-adjusted odds ratio (95% CI)	Denominators^2^
All sexually-active women aged 16–44 years	5.5% (4.8%-6.3%)			4980, 3481
***Sociodemographic factors***				
Age		*p*<0.001		
16–24	8.5% [7.1%-10.1%]	1.00		1678, 931
25–34	7.0% [5.8%-8.4%]	0.82 (0.61–1.08)		2245, 1250
35–44	2.0% [1.2%-3.3%]	0.22 (0.13–0.38)		1057, 1299
Sexual identity^3^		*p*<0.001	*p*<0.001	
Heterosexual	5.2% (4.5%-6.0%)	1.00	1.00	4780, 3344
Lesbian	10.6% (4.1%-24.6%)	2.18 (0.79–6.02)	2.47 (0.87–7.00)	60, 45
Bisexual	18.0% (12.0%-26.1%)	4.02 (2.44–6.62)	3.28 (1.95–5.51)	123, 79
Relationship status		*p*<0.001	*p*<0.001	
Married	2.0% (1.3%-3.2%)	1.00	1.00	1554, 1448
Cohabitating	6.4% (4.8%-8.5%)	3.29 (1.89–5.71)	2.83 (1.65–4.86)	879, 660
Previously married	2.9% (1.6%-5.4%)	1.44 (0.65–3.21)	1.52 (0.68–3.40)	280, 174
Single/never married	9.7% (8.4%-11.1%)	5.12 (3.12–8.41)	3.73 (2.24–6.23)	2259, 1193
Ethnicity		*p* = 0.005	*p* = 0.009	
White	5.9% (5.1%-6.8%)	1.00	1.00	4375, 3023
Mixed	9.8% (5.0%-18.3%)	1.73 (0.82–3.64)	1.46 (0.72–3.00)	142, 92
Asian/Asian British	1.1% (0.3%-4.3%)	0.18 (0.04–0.72)	0.19 (0.05–0.79)	238, 198
Black/Black British	1.7% (0.5%-5.5%)	0.28 (0.09–0.92)	0.28 (0.08–0.94)	157, 120
Chinese/other^4^	1.7% (0.4%-7.2%)	0.28 (0.07–1.25)	0.27 (0.06–1.21)	63, 44
Academic qualifications^5^		*p* = 0.038	*p* = 0.311	
None	4.0% (2.7%-6.0%)	1.00	1.00	415, 268
Qualifications typically gained at age 16	4.8% (3.6%-6.3%)	1.20 (0.71–2.02)	1.25 (0.74–2.09)	1674, 1173
Studying for/attained further academic qualifications	6.3% (5.4%-7.4%)	1.61 (1.03–2.53)	1.40 (0.89–2.21)	2625, 1869
***Health-related factors***				
Self-reported health status		*p*<0.001	*p* = 0.002	
Very good	3.9% (3.1%-4.8%)	1.00	1.00	2249, 1617
Good	7.1% (5.9%-8.6%)	1.92 (1.41–2.60)	1.83 (1.34–2.50)	2135, 1457
Fair	6.6% (4.8%-9.1%)	1.77 (1.16–2.71)	1.78 (1.16–2.73)	487, 334
Bad/very bad	6.0% (2.8%-12.6%)	1.60 (0.69–3.75)	1.65 (0.71–3.84)	109, 73
Treated for depression in the past year		*p* = 0.047	*p* = 0.006	
No	5.4% (4.7%-6.2%)	1.00	1.00	4581, 3216
Yes	7.6% (5.5%-10.5%)	1.45 (1.00–2.09)	1.69 (1.17–2.44)	397, 3216
Current smoking status		*p*<0.001	*p*<0.001	
No	3.0% (2.4%-3.8%)	1.00	1.00	3394, 2491
Yes	11.9% (10.1%-13.9%)	4.35 (3.23–5.86)	3.98 (2.94–5.39)	1586, 989
Current frequency of binge drinking^6^		*p*<0.001	*p*<0.001	
Never	1.9% (1.3%-2.9%)	1.00	1.00	1621, 1194
< Monthly	5.1% (4.0%-6.6%)	2.73 (1.68–4.44)	2.61 (1.60–4.24)	1273, 882
Monthly	7.2% (5.4%-9.5%)	3.92 (2.39–6.42)	3.40 (2.08–5.57)	902, 601
Weekly/daily	17.5% (14.3%-21.3%)	10.71 (6.62–17.31)	9.95 (6.11–16.19)	657, 438
***Sexual behaviours***				
Number of sexual partners^7^, past year		*p*<0.001	*p*<0.001	
1	3.8% (3.1%-4.7%)	1.00	1.00	3835, 2828
2	10.3% (7.4%-14.1%)	2.88 (1.88–4.24)	2.32 (1.49–3.60)	505, 295
3–4	14.3% (10.8%-18.6%)	4.18 (2.86–6.11)	3.16 (2.16–4.63)	391, 208
5+	18.4% (13.6%-24.5%)	5.67 (3.72–8.65)	4.15 (2.69–6.40)	219, 130
Same-sex partner(s), past year		*p*<0.001	*p*<0.001	
No	5.2% (4.5%-6.0%)	1.00	1.00	4798, 3360
Yes	14.5% (9.5%-21.5%)	3.08 (1.87–5.07)	2.87 (1.73–4.78)	181, 121
Number of partnerships^7^ without a condom, past year		*p*<0.001	*p*<0.001	
0	3.5% (2.2%-5.4%)	1.00	1.00	734, 501
1	4.3% (3.5%-5.2%)	1.23 (0.74–2.03)	1.41 (0.86–2.31)	3611, 2627
2+	18.3% (15.0%-22.2%)	6.19 (3.67–10.46)	5.24 (3.07–8.94)	575, 317
Used internet to find a sexual partner^7^ past year		*p*<0.001	*p*<0.001	
No	5.2% (4.5%-6.0%)	1.00	1.00	4804, 3374
Yes	15.4% (9.7%-23.6%)	3.29 (1.89–5.73)	3.46 (1.95–6.12)	174, 106

p-values from Wald test

^1^‘Recent illicit drug use’ defined as using drugs other than, or in addition to, cannabis in the past year. Excludes injected drugs.

^2^Unweighted, weighted denominators defined as participants aged 16–44 years reporting at least one sexual partner in the past year.

^3^Excludes those reporting ‘other’ to the question about sexual identity as this was reported by too few participants to provide robust estimates.

^4^‘Chinese’ and ‘other’ subcategories were merged because of small numbers in these categories.

^5^Limited to those aged at least 17 years.

^6^More than 6 units of alcohol on one occasion.

^7^Opposite-sex and/or same-sex sex partner(s).

Among women (but not men), self-reported general health was associated with reporting illicit drug use other than, or in addition to, cannabis, with prevalence higher in women describing their health as ‘bad/very bad’. Prevalence was also higher among men and women who reported receiving treatment for depression. Being a current smoker and/or reporting more regular binge drinking were also strongly associated with illicit drug use.

A positive association was observed between our measure of illicit drug use and each of the measures of recent sexual behaviour we investigated, including the number of sexual partners reported in the past year (aAORs: 5.03 for men and 4.15 for women reporting five or more partners vs. one partner), as well as reporting at least one same-sex partner, two or more sexual partners without a condom, using the internet to find sexual partners, and in men, reporting paying for sex, all in the past year (Tables 2 and 3).

### Sexual health outcomes associated with recent illicit drug use

After adjustment for age, sexually-active men and women aged 16–44 years who reported illicit drug use other than, or in addition to, cannabis were more likely to report sexual health clinic attendance and to have tested for chlamydia (both in the past year); these associations remained after additional adjustment for sexual identity and the number of partners reported during that time (Table 4). HIV testing followed a similar pattern but was more weakly associated. Men—but not women—who reported our measure of illicit drug use were also more likely to report having had STIs diagnosed. Among women, those reporting drug use were more likely to have had an unplanned pregnancy in the past year. This association remained after adjustment for age, sexual identity and partner numbers (AOR: 2.93).

**Table 4:** Variation in reporting key sexual health outcomes and STI/HIV risk perception according to recent illicit drug use^1^ status, by gender.

	Recent illicit drug use^1^ status:		Age-adjusted		Adjusted	
	Yes—reported recent illicit drug use % (95% CI)	No—did not report recent illicit drug use % (95% CI)	odds ratio for reporting outcome (95% CI)	*p*-value	odds ratio^2^ for reporting outcome (95% CI)	*p*-value
**Men**						
*Denominators*^3^:	*442*, *401*	*2953*, *3087*				
***Sexual health outcome***^4^:						
Sexual health clinic attendance	17.9% (14.3–22.1)	6.6% (5.8–7.5)	2.63 (1.95–3.56)	<0.001	1.79 (1.28–2.51)	0.001
Diagnosed with STI(s)	5.8% (3.9–8.7)	1.1% (0.8–1.5)	5.00 (2.83–8.84)	<0.001	3.10 (1.63–5.89)	0.001
HIV test	11.6% (8.7–15.5)	5.4% (4.5–6.4)	2.23 (1.53–3.26)	<0.001	1.48 (0.97–2.25)	0.069
Chlamydia test	29.2% (24.7–34.1)	15.1% (13.9–16.5)	1.97 (1.51–2.57)	<0.001	1.42 (1.06–1.92)	0.017
**Women**						
*Denominators*^3^:	*321*, *193*	*4659*, *3288*				
***Sexual health outcome***:^4^						
Sexual health clinic attendance	26.3% (20.7–32.7)	8.7% (7.8–9.6)	2.74 (1.93–3.89)	<0.001	1.99 (1.34–2.95)	<0.001
Diagnosed with STI(s)	2.7% (1.4–5.0)	1.6% (1.2–2.1)	1.16 (0.58–2.31)	0.670	0.75 (0.35–1.61)	0.465
HIV test	18.5% (13.9–24.2)	10.3% (9.3–11.3)	1.67 (1.15–2.41)	0.007	1.42 (0.97–2.07)	0.070
Chlamydia test	53.0% (45.8–60.1)	25.3% (23.9–26.7)	2.43 (1.79–3.30)	<0.001	1.94 (1.40–2.70)	<0.001
Unplanned pregnancy	5.3% (2.8–9.9)	1.5% (1.1–1.9)	3.05 (1.46–6.37)	0.003	2.93 (1.39–6.17)	0.005

p-values from Wald test

^1^‘Recent illicit drug use’ defined as using drugs *other than*, *or in addition to*, cannabis in the past year. Excludes injected drugs.

^2^Adjusted for age, sexual identity and numbers of sexual partners in the past year.

^3^Unweighted, weighted denominators defined as participants aged 16–44 years who reported sex in the year prior to interview for Natsal-3

^4^Sexual health outcomes experienced in the past year.

## Discussion

### Statement of main findings

Our study using data from a large, national probability survey shows that among the sexually-active British population aged 16–44 years, around one in ten men and one in twenty women reported having used drugs other than, or in addition to, cannabis in the past year. Use was considerably higher among those under 35 years so we adjusted for the confounding effect of age in our analyses of other factors we hypothesised to be associated with use. A number of other markers of poorer health, including binge drinking, smoking, and depression, remained associated, as observed elsewhere.[9,10,17,21,34,35] We found associations between our measure of illicit drug use and various sexual behaviours including higher numbers of partners, and importantly, having multiple partners with whom condoms were not used—behaviours strongly associated with STI transmission.[4] Illicit drug use was also more common among sexual minorities, both those not identifying as heterosexual and those reporting same-sex behaviour. This is in line with the many studies of gay men’s use of illicit drugs, [13,17,20,36,37] including but not exclusively in the context of ‘chemsex’ (the deliberate use of illicit drugs to facilitate engagement in sexual activities with one or more partners on a singular occasion).[18,38,39]. Our study also considered illicit drug use as an explanatory variable, and found that sexual health clinic attendance and chlamydia testing were more common among men and women reporting use, however, actual STI diagnoses were only found to be more commonly reported by men. Sexual health is broader than the absence of STIs, [40,41] and so this study also considered unplanned pregnancy as a marker of sexual health with potentially wider reaching impacts than STI diagnosis and clinic attendance. We observed an association between recent use of drugs other than, or in addition to, cannabis and unplanned pregnancy in the past year in women. We have also previously shown that ever experience of non-volitional sex, another adverse outcome with potentially long-term consequences, was more commonly reported by those who also reported illicit drug use.[42] These findings suggest that illicit drug use is thus an important risk factor for a number of adverse sexual health outcomes for the population as a whole. Given their implications, and the association between drug use and poor health more broadly, as discussed above, this suggests that holistic interventions are required.

### Strengths & weaknesses of the study

The design of Natsal-3 means that these findings can be considered as broadly representative of the British general population, and are not limited to particular groups, such as MSM (who have been the focus of many previous studies). However, a response rate of 57.7%, while in line with other major social surveys completed in Britain around the same time, [43,44] means that non-response could be a source of bias. We aimed to minimise this by weighting the sample so that it was broadly representative of the population according to the census with respect to gender, age, and geographical regions and our weighted sample was similar to the census with respect to ethnicity, health and marital status[24] however there may be other biases in the sample. Furthermore, the sampling strategy was limited to those living in private households, thereby excluding people living in institutions and the homeless, whose behaviour, and in particular, use of illicit drugs, may be different. Despite using CASI to encourage open and accurate reporting of behaviours that are widely regarded as socially-sensitive, and in the case of drug use, illegal, social desirability bias may have led to an under reporting of drug use. Although this may be less of an issue than in other surveys as it was just one of a number of socially censored behaviours that Natsal-3 asked about. This is perhaps reflected in the relatively low item non-response observed in Natsal-3 generally (typically 1–3%)[3] including for the questions about illicit drug use.

The cross-sectional design of Natsal means that causality and temporality cannot be determined, making it impossible to explore causal mechanisms through which drug use and sexual health behaviours and outcomes operate. Moreover, like other studies, Natsal-3 did not ask about drug use in the context of specific sexual occasions so it is unclear to what extent associations between drug use and sexual behaviours are at the level of the individual vs. a causal relationship of drugs being used concurrently with, and fostering, sexual risk behaviours. [45–47] Related to this, given the strong association seen between alcohol and drug use in our study and others [2,35] it is possible that the associations we observed between drug use and sexual behaviours are confounded by alcohol consumption. However, adjusting for current alcohol consumption did not affect associations between drug use and sexual health outcomes (data not shown). While use in the past year measures recent use, it cannot be considered as a measure of current use, nor as a proxy for regular use, neither of which Natsal-3 asked about.

In contrast to previous Natsal surveys, Natsal-3 asked about the use of nine different illicit drugs, and although the list is not exhaustive, with drugs such as ketamine, mephedrone and gammahydroxybutyrate/ gammabutyrolactone (GHB/GBL) [8,13,39] excluded, it did provide participants with the option of reporting ‘other non-prescribed drugs’. The question wording meant that it was not possible to determine the prevalence of use of specific drugs in the past year but we hope that including the data on ever use as S1 Web-Appendices will provide some insight for the interested reader. The inclusion of questions on illicit drug use in Natsal-3 reflects a key objective of this latest survey: to consider sexual health in the broader context of general health and well-being.[41,48] As such, our study benefitted from the wide range of data that were collected which has enabled us to investigate how illicit drug use varies with a number of sexual health as well as general health factors. As so many associations have been tested within this study we acknowledge that some significant ones may have arisen by chance. As we did not formally correct our *p*-values, we advise exercising caution in concluding association where *p*-values are less than 0.05 but greater than 0.01.

### Our findings in relation to other studies

Our prevalence estimates of illicit drug use were higher than those observed by the 2013/14 Crime Survey for England and Wales[2] even when we used the same criteria to define our numerator and denominator (aged 16–59 years, including cannabis in the definition and not limited to the sexually-active population): 18.4% (95% CI: 17.2%-19.6%) and 8.4% (7.7%-9.2%) among men and women, respectively in our data vs. 11.8% and 5.8% among men and women, respectively in the Crime Survey. Reasons for this are unclear. The Crime Survey also uses self-completion to collect these data but it is plausible that this difference reflects the framing of the surveys (crime vs. health) and/or potential reporting bias with Natsal-3 participants being more willing than those in the Crime Survey to disclose illicit drug use given the other sensitive behaviours that Natsal asked about.

Our findings of bi-directional relationships between illicit drug use, depression and STI diagnosis and sexual health clinic attendance in a general population sample are also consistent with previous studies in minority populations [14,15,49] and reinforce the need for holistic healthcare. This includes patient risk assessment that takes account of sexual risk as well as substance use, as per the British Association for Sexual Health and HIV (BASHH) guidelines, [50] which recommend that recreational drug history is incorporated into sexual history taking for all patients attending for STI screening. It also argues for the need for healthcare providers to be aware of trends in drug use in their locality, as well as their possible effects.

Much research has examined drug use in MSM, although much has focused on ‘chemsex’, and there is increasing recognition of the syndemic nature of MSMs health, of which drug use is only one component.[22] There is less research into WSW, who we also found to be more likely to report use of drugs, other than or in addition to, cannabis, particularly those self-identifying as bisexual. Bisexual women are also more likely to report sexual risk behaviour. [51]

The finding that both sexual health clinic attendance and testing for chlamydia were more common among people who had used drugs other than, or in addition to, cannabis may be regarded as positive outcomes. A recent analysis of data from sexual health clinics found that 7% of attenders had been under the influence of drugs before or during sex in the past three months.[52] Sexual health clinics may therefore be an appropriate setting to identify those who may benefit from drug treatment services and interventions that promote risk reduction practices in terms of sexual risk and illicit drug use, as well as mental well-being. While the increase in and promotion of remote STI testing may allow services to meet the needs of more individuals in a cost-effective way, it is important that sexual health services remain accessible to those with more complex needs.

Future research is needed to improve our understanding of the determinants of illicit drug use and of the contextual factors in which it occurs, including the interplay between illicit drug use and high-risk sexual behaviour, and the role of sexual pleasure within that relationship. More broadly, health promotion efforts should address illicit drug use alongside the use of other substances including alcohol and tobacco, and how their use relates to sexual and mental health risk behaviours and outcomes. Understanding these interactions will also be important for developing and delivering effective holistic interventions that mitigate against poor health outcomes that include, but are not limited to, drug use, thus maximizing individual and public health gain.

## Supporting information

S1 Web Appendices (DOCX)
