# Supplementary material for: Illicit drug use and its association with key sexual risk behaviours and outcomes: Findings from Britain’s third National Survey of Sexual Attitudes and Lifestyles (Natsal-3)
Source: PLoS One. 2017 May 18;12(5):e0177922. doi: 10.1371/journal.pone.0177922 (PMC5436851; doi:10.1371/journal.pone.0177922)
Supplement: S1 Web Appendices — (DOCX) [file pone.0177922.s001.docx]

**Web appendix A: Prevalence (95% CI) of *ever* using illicit drugs reported by sexually-active people in Britain aged 16-44 years by type of drugs used and age-group: Men**

|  | **Age-group** | | | **All** |  |
| --- | --- | --- | --- | --- | --- |
|  | **16-24** | **25-34** | **35-44** | **16-44** |  |
| *Weighted, unweighted denominators:* | *948, 1296* | *1238, 1378* | *1302, 721* | *3488, 3395* | *p*-value for |
|  | %  [95% CI] | %  [95% CI] | %  [95% CI] | %  [95% CI] | variation by  age-group |
| **Ever use of:** |  |  |  |  |  |
| Cannabis | 53.4% [50.3-56.5] | 54.7%  [51.8-57.6] | 45.5% [41.4-49.6] | 50.9%  [48.8-53.1] | <0.001 |
| Amphetamines | 11.7% [9.9-13.9] | 18.9%  [16.6-21.5] | 17.9% [15.1-21.2] | 16.6%  [15.1-18.2] | <0.001 |
| Cocaine or coke | 19.4% [17.0-22.1] | 28.7%  [26.1-31.5] | 17.4% [14.6-20.7] | 22.0%  [20.3-23.7] | <0.001 |
| Crack | 0.7%  [0.4-1.2] | 4.1%  [3.1-5.6] | 2.3%  [1.4-3.7] | 2.5%  [2.0-3.2] | <0.001 |
| Ecstasy | 17.3% [15.1-19.8] | 25.3%  [22.7-28.1] | 17.1% [14.2-20.4] | 20.1%  [18.4-21.8] | <0.001 |
| Heroin that was not injected | 0.4%  [0.2-0.8] | 2.3%  [1.6-3.3] | 2.0%  [1.1-3.3] | 1.7%  [1.2-2.3] | 0.003 |
| Acid or LSD | 8.1%  [6.5-10.1] | 14.2%  [12.3-16.3] | 14.8% [12.1-17.8] | 12.8%  [11.4-14.3] | <0.001 |
| Crystal Meth | 1.2%  [0.6-2.4] | 1.2%  [0.7-2.2] | 0.4% [0.07-2.0] | 0.9%  [5.8-1.4] | 0.25 |
| Amyl Nitrates | 6.8%  [5.3-8.5] | 12.6%  [10.8-14.7] | 11.7% [9.4-14.6] | 10.7%  [9.5-12.1] | <0.001 |
| Other non-prescribed drugs | 6.7%  [5.3-8.4] | 6.8%  [5.5-8.4] | 2.7%  [1.8-4.1] | 5.3%  [4.5-6.2] | <0.001 |
|  |  |  |  |  |  |
| **Any illicit drug use, ever** | 56.2% [53.1-59.2] | 58.1%  [55.1-61.0] | 49.1% [45.0-53.3] | 54.2%  [52.1-56.4] | 0.003 |
| Only ever cannabis use | 28.4% [25.6-31.4] | 22.0%  [19.5-24.6] | 21.2%  [18.2-24.5] | 23.4%  [21.7-25.2] |  |
| Ever use of both cannabis & other drugs | 25.0% [22.4-27.8] | 32.7%  [30.0-35.6] | 24.3%  [21.0-28.0] | 27.5%  [25.7-29.4] |  |
| Ever used drugs but not cannabis | 2.8%  [2.0-3.9] | 3.4%  [2.6-4.5] | 3.6%  [2.5-5.3] | 3.3%  [2.7-4.1] |  |
|  |  |  |  |  |  |

**Web appendix B: Prevalence (95% CI) of *ever* using illicit drugs reported by sexually-active people in Britain aged 16-44 years by type of drugs used and age-group: Women**

|  | **Age-group** | | | **All** |  |
| --- | --- | --- | --- | --- | --- |
|  | **16-24** | **25-34** | **35-44** | **16-44** |  |
| *Weighted, unweighted denominators:* | *931, 1678* | *1250, 2245* | *1299, 1057* | *3481, 4980* | *p*-value for |
|  | %  (95% CI) | %  (95% CI) | %  (95% CI) | %  (95% CI) | variation by age-group |
|  |  |  |  |  |  |
| **Ever use of:** |  |  |  |  |  |
| Cannabis | 41.3% [38.7-44.0] | 38.5% [36.2-40.9] | 31.2% [28.2-34.4] | 36.6% [35.0-38.2] | <0.001 |
| Amphetamines | 6.9%  [5.7-8.3] | 10.7% [9.3-12.3] | 11.4% [9.4-13.8] | 10.0% [9.0-11.0] | <0.001 |
| Cocaine or coke | 13.6% [11.9-15.5] | 16.3% [14.6-18.2] | 7.9%  [6.3-9.9] | 12.4% [11.4-13.6] | <0.001 |
| Crack | 0.7%  [0.4-1.3] | 0.9%  [0.6-1.4] | 0.3% [0.1-0.7] | 0.6%  [0.5-0.9] | 0.050 |
| Ecstasy | 10.2% [8.6-11.9] | 13.0% [11.5-14.6] | 9.1%  [7.4-11.2] | 10.8% [9.8-11.8] | 0.007 |
| Heroin that was not injected | 0.5%  [0.2-1.0] | 0.6%  [0.3-1.0] | 0.8%  [0.4-1.5] | 0.6%  [0.4-0.9] | 0.58 |
| Acid or LSD | 2.4%  [1.7-3.2] | 4.5%  [3.6-5.5] | 6.9%  [5.3-8.8] | 4.8%  [4.1-5.6] | <0.001 |
| Crystal Meth | 0.5%  [0.3-1.0] | 0.5%  [0.2-0.9] | 0.1% [0.03-0.5] | 0.4%  [0.2-0.6] | 0.13 |
| Amyl Nitrates | 3.2%  [2.4-4.2] | 4.1%  [3.2-5.1] | 3.8% [2.7-5.3] | 3.7%  [3.1-4.4] | 0.51 |
| Other non-prescribed drugs | 1.7%  [1.1-2.5] | 2.2%  [1.6-3.0] | 0.7%  [0.4-1.4] | 1.5%  [1.2-1.9] | 0.004 |
|  |  |  |  |  |  |
| **Any illicit drug use, ever** | 43.3% [40.6-45.9] | 43.0% [40.6-45.5] | 33.8% [30.7-36.9] | 39.6% [38.0-41.3] | <0.001 |
| Only ever cannabis use | 24.8% [22.6-27.2] | 20.4%  [18.5-22.5] | 17.2% [14.9-19.6] | 20.4% [19.1-21.7] |  |
| Ever use of both cannabis & other drugs | 16.5% [14.6-18.6] | 18.1% [16.4-20.0] | 14.1% [11.9-16.5] | 16.2% [15.0-17.4] |  |
| Ever used drugs but not cannabis | 1.9%  [1.3-2.8] | 4.5%  [3.7-5.5] | 2.6%  [1.7-3.7] | 3.1%  [2.6-3.6] |  |
|  |  |  |  |  |  |
